# Extreme Oncoplastic Surgery for Multifocal/Multicentric and Locally Advanced Breast Cancer

**DOI:** 10.1155/2019/4262589

**Published:** 2019-02-20

**Authors:** Chaitanyanand B. Koppiker, Aijaz Ul Noor, Santosh Dixit, Laleh Busheri, Gautam Sharan, Upendra Dhar, Hari Kiran Allampati, Smeeta Nare

**Affiliations:** ^1^Orchids Breast Health Clinic, Prashanti Cancer Care Mission, 1&2, Kapilavastu, Senapati Bapat Road, Pune, Maharashtra 411016, India; ^2^Department of Radiation Oncology, Inlaks and Budhrani Hospital, Pune 411001, India; ^3^Ruby Hall Clinic, Pune, Maharashtra 411040, India

## Abstract

**Introduction:**

Breast conserving surgery (BCS) followed by radiation therapy (RT) has become the preferred alternative to mastectomy for patients with early stage breast cancer (BC). Randomized trials have confirmed equivalent locoregional control and overall survival for BCS and mastectomy. Extreme Oncoplasty (EO) extends the indications of BCS for patients who would otherwise require mastectomy, ensuring better aesthetic outcomes and oncological safety.

**Methods:**

BC patients with multifocal/multicentric (MF/MC) tumors, extensive DCIS, or large tumor >50mm underwent EO at our breast unit. Therapeutic reduction mammaplasty (TRM) with wise pattern preoperative markings and dual pedicle technique involving parenchymal rearrangement was used for oncoplastic reconstructions in majority of the cases followed by RT. Patient reported outcome measures (PROMs) were assessed using the validated Breast-Q questionnaire.

**Results:**

Of the 39 patients in the study, 36 had unilateral and 3 had bilateral BC. Mean age was 47.2 years. Median tumor size was 75mm. 17 (43.6%) patients received NACT; none achieved a complete clinical response. 28 (71.8%) patients were administered to adjuvant chemotherapy. 33(84.6%) patients received RT to the breast with a median dose of 50Gy in 28 fractions and a boost dose of 10Gy in 5 fractions to the tumor bed. No major complications or local recurrences were observed. Excellent Breast-Q scores were observed in patients undergoing EO after 12 months of follow-up.

**Conclusion:**

EO followed by RT results in acceptable local-regional control, low rate of complications, and high patient satisfaction. In selected patients, EO could provide a safe alternative for breast conservation surgery instead of mastectomy.

## 1. Introduction

Breast conservation therapy (BCT) which includes breast conservation surgery (BCS) and adjuvant radiation therapy (RT) is a standard protocol to achieve high local control with acceptable aesthetic outcomes [1–3]. Multiple prospective randomized trials [4], some with a follow-up of 20 years, have established equivalent survival rates between mastectomy and breast conservation surgery (BCS) with negative margins. Recent studies from various countries including India indicate that women treated with BCT have a significant survival advantage when compared to mastectomy [5–7].

At present, breast surgeons are attempting to extend the scope of breast conservation so as to include scenarios which are otherwise contraindicated for BCS particularly in multicentric (MC) or multifocal (MF) tumors. Clinical management of MF/MC breast tumors is a challenge since the choice of optimal surgical approach is controversial. Previously, mastectomy was considered as the standard choice [8–10]. However, significant local recurrence rates (2.5% to ~40%) have limited the utility of mastectomy in such scenarios [11–15].

In contrast, breast cancer (BC) patients with large T2 and T3 tumors (size range: 40-120mm) who underwent BCT were shown to have acceptable cosmesis without compromising locoregional control or survival [16]. In the last decade, oncoplastic breast surgery (OBS) has emerged as an integrated approach to achieve optimal oncological outcomes and cosmesis. This approach allows tumor excision with wider margins during BCS without compromising the aesthetic outcome [17]. In addition, OBS has shown comparable oncological efficacy with conventional BCS in achieving adequate surgical margins and recurrence rates [18–21].

In recent years, the technique of “Extreme Oncoplasty” (EO) has emerged as a promising option in selective patients with adequate breasts (cup size ≥ C) where in BCS is possible inspite of large volume resections [22]. Indeed, EO could be used to conserve breasts in scenarios for which mastectomy would be have been the treatment of choice offered by most surgeons. These situations include (a) tumor size >50 mm, (b) MF and MC tumors, (c) extensive ductal carcinoma in situ (DCIS), (d) extensive intraductal component > 50 mm, (d) previously irradiated breast with a new or recurrent cancer within the same breast, (e) a locally advanced breast carcinoma with limited or partial imaging response to neoadjuvant chemotherapy (NACT), and (f) patient with excision biopsy with inappropriate scar [22, 23]. Thus, EO provides an alternative to mastectomy, extends the scope of breast conservation, provides better clinical outcomes, and improves quality of life (QoL) [22].

There are very few reports in the literature which describe the application of the EO technique in BC management. Therefore, the aim of this study is to analyze the clinical, postsurgical, and patient satisfaction outcomes in an EO study cohort at our breast unit. In this report, we describe our experience in a series of 39 BC patients who underwent EO using wise pattern/vertical scar therapeutic reduction mammaplasty (TRM) technique.

## 2. Material and Methods

### 2.1. Surgical Technique

Our EO surgery technique encompasses excision of large volume of the breast dictated by the extent of tumor. This is facilitated by the technique of Therapeutic Reduction Mammoplasty (TRM) which is performed either by a wise pattern and/or vertical scar skin pattern. In some patients, the central wedge excision with immediate nipple reconstruction (wise pattern) and in few patients nipple areolar grafting was performed. In exceptional cases, lateral mammaplasty techniques were used.

The surgery begins by marking out the wise pattern. The next step is to localize the tumor and excise it with wide margins by going through one of the limbs of the wise pattern. This localization is performed either preoperatively by stereotactic guide-wire placement or by placement of wire and needle on the operating table using a high resolution ultrasonography. The tumor and its quadrant are then widely excised and further imaging of the specimen is performed using specimen mammography to ensure that the tumor is excised with wide margins. After this step, the shaved margins of the cavity are further excised and sent for frozen sections. Once clarity about the tumor margins of the excision cavity is achieved, the decision is made to use one of the pedicles for carrying the nipple areolar complex (NAC) and filling in the defect by appropriate mobilization of the internal local breast flaps.

Most of the cases in our study cohort have been operated using the dual pedicle technique. NAC was carried on superior pedicle and the inferior pedicle was used to fill the defect caused by excision of the tumor.

### 2.2. Patient Selection

This is a single institutional study involving retrospective analysis of prospectively collected data. During the study duration, a total of 42 BC patients with large breasts having either MF/MC or large tumor (>50 mm) or previous oddly placed large scar or extensive DCIS were included. All patients were initially recommended to have a mastectomy but agreed to breast conservation after appropriate counseling by the breast oncoplastic surgeon. For the purpose of this study, large breast is defined as the one with a cup size of C or larger. Written informed consent was obtained from all patients for collection of study-relevant medical data inclusive of clinical management and routine follow-up visits. Data collection included demography, medical history, clinicopathological characteristics, details of adjuvant therapy, surgical intervention, postsurgery complications, and follow-up details.

Out of these 42 patients, 39 completed one-year postsurgery follow-up and were analyzed for surgical outcomes and patient reported outcome measures (PROMs).

### 2.3. Clinical Management

BC diagnosis was based upon clinical examination and radiological evaluation of breast and axilla using Full Field Digital Mammography (FFDM) with 3-D Tomosynthesis (Siemens Mammomat Inspiration™) and Ultrasonography (Siemens Acuson S2000™). Histopathological studies on Tru-Cut biopsy samples (majority of cases) or vacuum assisted biopsy (for index tumors, Encor Ultra™) samples were performed for confirming diagnosis of breast carcinoma. Similarly, ultrasonography and fine needle aspiration cytology were used for investigating axillary lymph node metastasis. Confirmed BC cases underwent EO surgery at a network hospital site. The oncologic management with chemoradiation protocols was undertaken by a multidisciplinary clinical team in accordance with the current NCCN guidelines.

### 2.4. Radiation Therapy

The Radiation Therapy (RT) dose planning was aimed at achieving a Biologically Effective Dose (BED) of 50 Gy for all patients. The breast along with the supraclavicular region (if indicated) was irradiated by 6 MV photon beams using Forward Plan Field-in-Field Intensity Modulated Radiation Therapy (F-P FiF IMRT). Two tangential fields along with multiple subfields were used for this treatment. CT images (5-mm thickness) were obtained at different transverse sections covering the region of interest with adequate margins to create a 3D image. Volume delineation (CTV, Contralateral breast, Lung and Heart) on CT images was performed on an Eclipse™ contouring station. Computerized treatment planning was performed on Eclipse™ treatment planning system (TPS) (Version 13.5.35). High energy linear accelerator (Elekta Synergy, Elekta Medical System™, UK) with 80 leaves Multileaf Collimator (MLCi) was used for tangential treatments to the breast field. RT plan was accepted if at least 95% of prescribed dose covers the 100% of planning target volume (PTV). Hot spot in PTV was accepted up to 110% of prescribed dose. Tumor bed boost, wherever indicated, was performed using either an electron portal or Simultaneous Integrated Boost (SIB) technique.

### 2.5. Study Assessments

Postsurgery outcomes were assessed by oncosurgeons. Complications such as hematoma, seroma, infection, skin necrosis, nipple necrosis, and wound dehiscence were recorded. Complications were classified as “major” when they required surgical intervention and “minor” when they were managed conservatively. We also noted the time between completion of the surgery and start of the adjuvant therapy to ascertain any delays in the adjuvant therapy.

### 2.6. PROMs

The Patient Reported Outcomes Measures (PROMs) were used to evaluate patient satisfaction and quality of life (QoL) after EO. To assess PROMs, a standardized Breast-Q questionnaire was utilized. The Breast-Q module was divided into multiple independent scales. Higher scores indicate greater patient satisfaction and functionality [24].

## 3. Results

### 3.1. Representative Case Study

**Figure 1:**
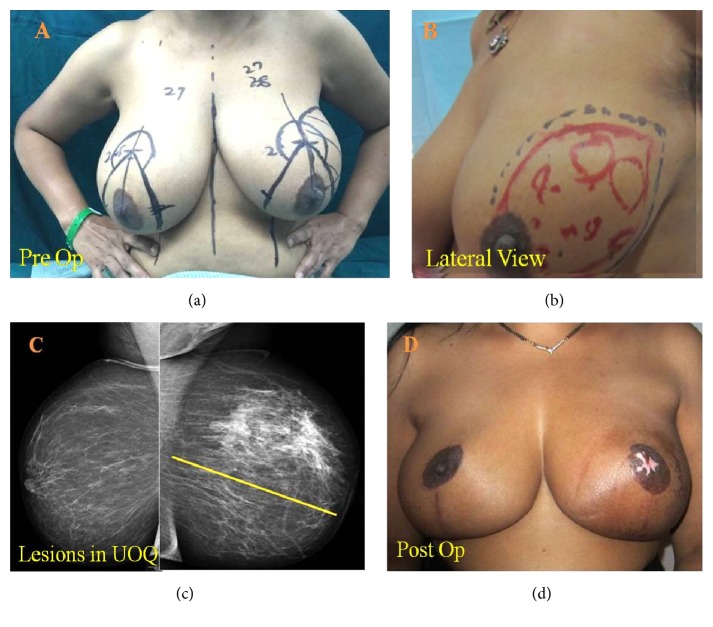
*Representative Case Study*. (a) Preoperative images. (b) Lateral view shows skin and breast tissue that will be removed. (c) Mammography shows lesion in UOQ. (d) Postoperative images.

A 40-year-old patient with E-cup breasts presented with a large diffuse lump in the left upper outer quadrant (UOQ). Mammogram revealed a MF/MC tumor that extended from 3 o' clock position to 12 o' clock position measuring 11.8 x 7.9 mm. Tru-Cut biopsy suggested DCIS of intermediate grade. Immunohistochemistry revealed ER (Estrogen Receptor)/PR (Progesterone Receptor) positive status. The patient was initially advised mastectomy but insisted on conserving her breast and sought a second opinion. Hence, TRM was planned. The tumor was localized with localization wires under mammography guidance. The patient was marked for wise pattern incision and excision of the whole UOQ was performed. Specimen mammography was performed to confirm complete removal of tumor. Shave margins were sent for frozen section evaluation and were reported negative. The skin over lower, medial, lateral, and superomedial quadrants was mobilized in the mastectomy plane and other quadrants were used to fill the defect. Since the dissected sentinel node was positive, further axillary dissections were performed through the same incision. 2/16 nodes were positive. Though the tumor location was close to the nipple, the nipple core and margins of the NAC were negative for DCIS on frozen sections. The breast tissue was reshaped and reconstructed. The NAC was used as a nipple-areola graft. The post-op histopathology revealed Grade II IDC with extensive DCIS and a lesion spanning 75 mm in UOQ. The patient received adjuvant RT, followed by electron boost to the tumor bed. The patient was counseled for adjuvant therapy and chose to have adjuvant endocrine therapy. The patient tolerated treatment well and is disease-free after 4 years post-diagnosis. The pre- and postsurgery images for this patient are depicted in Figure 1. The maximum and mean doses of RT received by the structures are tabulated in Supplementary Table 1.

### 3.2. Study Cohort Demography

Out of 42 EO cases, 39 patients with large breasts who had completed 12 months post-surgery follow-up were included in the study. 36 patients had unilateral and 3 had bilateral BCs. Tumors were found in the upper outer quadrant of the breast in 22 (56.4%) patients, lower outer quadrant in 06 (15.4%) patients, and lower inner quadrant in 06 (15.4%) patients. The mean age of patients at diagnosis was 46.3 years. Majority of these patients were at stages II or III. Most of the diagnosed BC cases were IDC and/or DCIS. 17 (43.6%) patients received NACT; none achieved a complete clinical response. In addition, adjuvant therapy was administered to 71.8% of patients. In the postoperative period, adjuvant chemotherapy and/or RT was administered to patients according to clinical indications. 32 (82.05%) patients received RT (Table 1).

Demographic distribution of study participants and their clinicopathological characteristics are summarized in Table 1. The median tumor size was 75mm. Clear margins were achieved in all the patients on frozen section as well confirmed on final histopathology analysis. The average margin achieved distance was 5mm. No local recurrence was observed (Table 2). None of the patients in our study cohort experienced any delays in their adjuvant therapies.

### 3.3. Postoperative Outcomes

There were no major postoperative complications (defined as requiring inpatient hospitalization or returning to the operating theatre). There were 3 cases of minor complications including 1 case of seroma and 2 cases of minor wound healing. All of them were treated conservatively in the outpatient settings (Table 3).

### 3.4. Radiation Therapy

Of the 39 patients included in this study, 32 patients underwent RT as clinically indicated. It was not possible to obtain RT-related data from the remaining 7 patients as they were lost to follow-up. While 22 of these 32 patients received RT to the whole breast alone, 10 received RT to the breast along with the supraclavicular region. Out of 32 patients, 18 patients had left-sided lesion and 14 patients had right-sided lesion. Forward Plan Intensity Modulated Radiation Therapy (IMRT) was used to treat 27 patients. 4 of the 5 patients who were treated using Volumetric Modulated Arc Therapy (VMAT) also received Simultaneous Integrated Boost (SIB). (Supplementary Table 2)

### 3.5. PROMs

PROMs data was collected from the study participants after 12 months postsurgery with the Breast-Q questionnaire. Out of 39 study participants, 29 (74.3%) responded to the questionnaire. These PROMs were used to assess patient reported satisfaction and QoL after EO. The results indicated improvement in the following four areas evaluated after surgery. The mean scores for BREAST-Q scales (±standard deviation) were Satisfaction with Breasts (78.0±16.6), Satisfaction with Outcome (85.7±13.7), Psychosocial Wellbeing (90.8 ± 11.5) and Sexual Wellbeing (75.8±11.7) (Table 4).

## 4. Discussion

BCT is considered a standard treatment option for selective BC patients with acceptable oncological safety, improved aesthetic outcomes, and better survival. In patients with large tumors, BCT is feasible if excision margins are free of tumor and an acceptable cosmesis can be obtained [3, 4, 16]. Results from NSABP-06 and EORTC-10801 trials have indicated that BCT is safe for patients with tumors >20mm [4, 25].

OBS allows tumor excision with wider resection margins and acceptable oncological and aesthetic outcomes [26]. This approach can achieve higher rates of negative margins with low local recurrence and better cosmesis. OBS has been shown to have a positive impact on QoL and self-esteem of patients. [27]. OBS technique namely TRM allows wide excision, reduced margin positivity, and better aesthetic outcome compared to conventional BCS [28–30]. Therefore, the indications for conventional BCS can be extended further for large tumors (>40mm), central quadrant and MF/MC tumors and extensive DCIS. Indeed, TRM is considered as a suitable option for OBS, especially, in women with large breasts on which wide resections can be performed [31, 32].

Given the increased risk of local recurrence, defining appropriate indication(s) for choosing BCS over mastectomy in patients with MC/MF tumors is important [8–10]. However, current indications are ambivalent. Local recurrence rates and overall survival after BCS did not differ in patients with unifocal or multifocal tumors [33]. In contrast, in selected MF/MC cases, BCS followed by adjuvant therapy was shown to be a safe alternative to mastectomy if negative surgical margins were achieved during surgery [14]. Furthermore, a recent study comparing oncological outcomes after mastectomy or BCS in patients with unifocal or MF/MC tumors shows significant increase in local recurrence rates after mastectomy [15]. Recently, the EO technique introduced by Silverstein et al. has demonstrated successful BCS outcomes and improvement in QoL in patients with MF/MC and large tumors (≥ 50mm). With this technique, the rate of local recurrence was comparable in patients with MF tumors that underwent either EO or conventional BCS.

In our study, we report the oncoplastic outcomes after EO in a single institutional cohort of 39 patients with large breasts (cup size ≥ C) with MC/MF or large tumors (≥50mm). While no major complications were observed after EO procedure, a low rate of minor complications (7.7%) was observed. The PROMs in these patients indicate better acceptance of the EO procedure with improvement in QoL. Breast-Q parameters such as satisfaction with breast, satisfaction with outcome, psychosocial wellbeing, and sexual wellbeing showed a positive trend after EO.

RT is an integral component of BCT and improves locoregional tumor control along with disease-free and overall survival [34–36]. However, RT increases the risk of postoperative complications such as severe capsular contracture in implant-based breast reconstructions [37–39]. Alternatively, autologous reconstruction can be a feasible option for patients that require PMRT [39]. However, this surgery type is associated with significant morbidity, complication, and longer hospital stay. Therefore, choice of optimal RT technique is critical in OBS to ensure satisfactory oncological outcomes with minimal postoperative complications.

Conventionally, a RT plan encompasses a total dose of 45-50 Gy in 25 daily fractions over 5 weeks followed by a boost of 10-16 Gy in 5-8 fractions. Nowadays, hypofractionated RT (dose >2 Gy per fraction) delivered in fewer fractions over a shorter treatment period is an alternative option [40]. In addition, a tumor bed boost delivered to selective patients by an electron beam of specific energy after whole breast irradiation (WBI) has been shown to improve local control, particularly, in patients <40 years [41]. Currently, newer RT techniques such as Simultaneous Integrated Boost (SIB) have been introduced to deliver boost dose concomitant to WBI, thereby reducing skin toxicity and fibrosis [42]. Indeed, in our study cohort, the 4 EO patients who received an SIB-IMRT did not develop any major complications. This observation indicates the feasibility of using SIB-IMRT for in patients who have undergone EO surgery.

Despite the promising results related to post-EO surgery outcomes and RT-associated complications, our study has a few limitations. This study only describes data from single breast unit with small number of patients (n = 39) with a 12-month postsurgery follow-up. To overcome these limitations, we are continually recruiting patients to increase the sample size and ensure long-term follow-up. In future, a multicentric study will be needed to avoid investigator bias.

## 5. Conclusion

The preliminary results of our study indicate that selective patients (with MF/MC or large tumors) who were initially considered for mastectomy can be alternatively treated using EO followed by RT (with an optional boost to tumor bed, if indicated). EO can be considered as a safe and feasible surgical option for such patients without compromising aesthetic outcomes.

## Figures and Tables

**Table 1:** Demography and clinicopathological profile of study participants.

Characteristics (n=39)		Number, %
Age, years	<=35	04 (10.2%)
	36-50	23 (58.9%)
	>51	12 (30.7%)

Tumor Location	Upper Inner Quadrant	05 (15.4%)
	Upper Pole	Nil
	Upper Outer Quadrant	22 (56.4%)
	Central	02 (5.1%)
	Lower Outer Quadrant	06 (15.4%)
	Lower Pole	01 (2.5%)
	Lower Inner Quadrant	06 (15.4%)
	Others	01(2.6%)

Type of Tumor	DCIS	02 (5.1%)
	IDC	26 (66.7%)
	IDC + DCIS	07(17.9%)
	Others	4 (10.3%)

Receptor Status	ER Positive	26 (66.7%)
	PR Positive	20 (51.3%)
	Her-2 Positive	13 (33.3%)
	TNBC	06 (15.14%)

Grade*∗*	I	1 (2.6%)
	II	26 (66.7%)
	III	08 (20.5%)

Stage*∗*	0	1 (2.6%)
	I	3 (7.7%)
	II	18(46.1%)
	III	12(30.7%)

NACT	-	17(43.6%)

Adjuvant Chemotherapy	-	28 (71.8%)

RT	-	32 (82.05%)

*∗*Data for all 39 patients could not be obtained due to being lost to follow-up.

**Table 2:** Salient features of EO cohort.

Characteristics(n =39)	Extreme (>50mm)
*N*	39

Mean Age	46.3

Mean Volume	432.8 cc

Median Span	75 mm

NACT	17/39 (43.6%)

Margins (0.1 – 0.9mm)	Nil

Margins (>1 mm)	39/39 (100%)

Average Margin Distance	5 mm away (approximately)

Re-excision	Nil

Mastectomy	Nil

Any Local Recurrence	Nil

Follow-Up	12 months

**Table 3:** Summary of postoperative complications.

Characteristics		Complications Number, % (n = 39)
Major Complications	Hematoma (requiring surgical evacuation)	Nil
	Infection (requiring surgical drainage/debridement under general anesthesia	Nil
	Skin Necrosis (requiring surgical debridement under general anesthesia)	Nil
	Nipple Necrosis (requiring surgical debridement/complete nipple loss)	Nil
	Wound Dehiscence (requiring return to theatre for resuturing)	Nil

	*Total*	Nil

Minor Complications	Seroma (requiring aspiration)	1 (2.6%)
	Hematoma (managed conservatively)	Nil
	Infection (requiring antibiotics)	Nil
	Skin Necrosis (managed conservatively by dressings)	Nil
	Nipple Necrosis (managed conservatively by dressings)	Nil
	Wound Dehiscence (managed conservatively)	2 (5.1%)

	*Total*	*3 (7.7%)*

**Table 4:** PROMs from EO study participants.

S. No.	Breast-Q Parameters	Mean±S.D.(*n* = 29)
1.	Satisfaction with Breast	78.0 ±16.6

2	Satisfaction with Outcome	85.7 ±13.7

3.	Psychosocial Wellbeing	90.8 ± 11.5

4.	Sexual Wellbeing	75.8 ± 11.7

## Data Availability

The figures and tables used to support the findings of this study are included within the article. The figures and tables used to support the findings of this study are included within the supplementary information file(s). The medical records and related data used to support the findings of this study are restricted by the Independent Ethics Committee (IEC) of the parent institute (Prashanti Cancer Care Mission) in order to protect the patient privacy. Data will be available from corresponding author upon request after approval of the IEC.

## Abbreviations (Arranged Alphabetically)

BC: Breast Cancer
BCS: Breast Conservation Surgery
BCT: Breast Conservation Therapy
DCIS: Ductal Carcinoma In Situ
EO: Extreme Oncoplasty
IDC: Intraductal Carcinoma
MF/MC: Multifocal/Multicentric
NAC: Nipple Areolar Complex
OBS: Oncoplastic Breast Surgery
PROMs: Patient Reported Outcome Measures
QoL: Quality of Life
RT: Radiation Therapy
SIB: Simultaneous Integrated Boost
TRM: Therapeutic Reduction Mammaplasty
UOQ: Upper Outer Quadrant.

## Supplementary Materials

Supplementary Materials Supplementary Table 1: Dose Received by Different Structures (for Representative Case Study). Supplementary Table 2: RT data for EO Cohort.
