# Supplementary material for: Extreme Oncoplastic Surgery for Multifocal/Multicentric and Locally Advanced Breast Cancer
Source: Int J Breast Cancer. 2019 Feb 20;2019:4262589. doi: 10.1155/2019/4262589 (PMC6402217; doi:10.1155/2019/4262589)
Supplement: Supplementary Materials — Supplementary Table 1: Dose Received by Different Structures (for Representative Case Study). Supplementary Table 2: RT data for EO Cohort. [file 4262589.f1.pdf]

Supplementary Table 1: Dose Received by Different Structures (For Representative Case Study)

Structure	Volume , cc	Maximum Dose, cGy	Mean Dose, cGy
Planning Target Volume	1041.8	4955.7	4682.0
Breast, Contralateral	1028	1074.7	67.4
Heart	632.2	4440.0	693.2
Lung, Ipsilateral	1104.6	4536.9	1694.6
Lungs	2420.2	4536.3	923.0

Supplementary Table 2: RT data for EO Cohort

Features	Number
Patients who received RT to whole breast	22
Patients who received RT to whole breast + SCF	10
Patients with left sided lesion	18
Patients with right sided lesion	14
Patients treated by F-P FiF IMRT	27
Patients treated by VMAT	5
Patients who were given SIB	4
Patients who were given electron boost	27
Patients who were not given electron boost/ SIB	1
